# Physical activity during COVID-19 induced lockdown: recommendations

**DOI:** 10.1186/s12995-020-00278-9

**Published:** 2020-08-12

**Authors:** Eszter Füzéki, David A. Groneberg, Winfried Banzer

**Affiliations:** Division of Preventive and Sports Medicine, Institute of Occupational, Social and Environmental Medicine, Goethe University Frankfurt, Theodor-Stern-Kai 7, Haus 9B, 60590 Frankfurt am Main, Germany

**Keywords:** Physical activity, Lockdown, Health

## Abstract

Measures aiming at containing the Coronavirus disease 2019 (COVID-19) include isolation, social distancing, and quarantine. Quarantine and other lockdown instruments show promise in reducing the number of COVID-19 infections and deaths. It is reasonable to assume that lockdown leads to reduced levels of physical activity in the general population. Potential detrimental health effects of lockdown, such as psychological distress and physical inactivity induced maladaptations must be addressed. The current review summarizes harmful effects of limited physical activity on mental and physical health due to social distancing and quarantine and highlights the effects of simple physical activity regimes counteracting these detrimental effects, with a special emphasis on acute effects.

## Background

The Coronavirus disease 2019 (COVID-19), caused by a novel coronavirus, SARS-CoV-2 (severe acute respiratory syndrome coronavirus-2) has reached pandemic dimension worldwide. In the absence of a preventive vaccine and specific pharmaceutical options, public health measures are essential to contain the spread of the virus. Successful strategies have already been identified; beyond strict hygienic rules these measures include isolation, social distancing, and quarantine. A rapid Cochrane systematic review, based primarily on simulation studies, indicates that quarantine, especially if combined with other measures, such as school closures, travel restrictions and social distancing might reduce the number of COVID-19 infections and deaths [1]. A report from China confirms these findings [2]. Mathematical modelling projecting potential scenarios for SARS-CoV-2 transmission in the future foresee that prolonged or intermittent social distancing may be necessary into 2022 [3].

According to a BBC News report, dated 07.04.2020 over 100 countries worldwide have implemented full or partial lockdown measures as of late March, affecting billions of citizens [4]. Severity of measures differ, but they all aim at limiting social contacts, which necessarily implies opportunities to move around.

While quarantine and social distancing measures seem indispensable as of now, possible detrimental social, psychological, health and economic consequences must also be considered. Psychological impact of quarantine can be wide-ranging, substantial and potentially sustained [5]. Symptoms include emotional disturbance, depression, stress, low mood, irritability, insomnia, post-traumatic stress symptoms and anxiety [5]. Little is known about the psychological effects of less rigorous, but prolonged measures of confinement.

The aim of the present article is 1) to review harmful effects of limited physical activity on mental and physical health due to social distancing and quarantine and 2) to highlight the effects of simple physical activity regimes, with a special emphasis on acute effects. We provide recommendations for evidence based physical activities that are feasible even in lockdown situations, and that hold promise to help patients cope with the situation. This knowledge can build the base for physical activity counseling in COVID-19 lockdown times.

## Metabolic, vascular and musculoskeletal effects of reduced physical activity

Current physical activity recommendations advise the general population to aim at performing at least 150–300 min of moderate or 75–150 min of vigorous physical activity a week (or a combination of these), and muscle strengthening activities at least twice a week [6]. It is however essential to emphasize that physical activity much below this level is also beneficial, and that evidence based health enhancing physical activity can come in many easily available shape or form [7].

It is reasonable to assume that lockdown measures have fundamentally changed work and transport related physical activities for a large proportion of the working population. With the closure of sports and fitness clubs as well as outdoor exercise facilities also leisure time activities might be affected, potentially leading to a further decline of already low levels of physical activity at the population level [8].

Step reduction studies, i.e. intervention studies in which participants are instructed to limit the number of steps taken, which might best mimic current situation for large parts of the general population under lockdown, shed light on the health effects of even relatively short periods of physical inactivity [9, 10].

The reduction of ambulatory activity from a relatively high (about 10.000 steps/day, which approximates recommended level of physical activity) to a low level (less than 2500 steps/day) for 14 days leads to metabolic maladaptations, such as increased intraabdominal and ectopic fat accumulation, hyperinsulinaemia even in young healthy adults [9, 10]. Further, this short term reduced activity results in an up to 6.6% ml/min/kg loss of cardiorespiratory fitness, and muscle atrophy in the lower extremities [9]. Fourteen days of step reduction in elderly participants induces detrimental alterations in glucose and insulin metabolism, impaired skeletal muscle protein synthesis, losses in muscle mass, as well as rise in inflammatory cytokines [9, 10].

Halving daily step count (from about 10.000 to 5.000) for just 5 days also led to a marked reduction in endothelial function at the popliteal but not brachial artery and a fivefold increase in endothelial microparticles in young healthy participants [11].

Importantly, resuming higher levels of ambulatory activities can reverse negative effects [9], and concomitant (even low intensity) resistant exercise (i.e. resistance exercise while ambulatory activity is kept reduced) can contribute to preservation of anabolic and insulin sensitivity [12]. This reversal, however, might be incomplete or require longer and more intensive activity periods in the elderly and chronically ill than in younger healthy adults [9, 10, 13].

Taken together, even acute, short periods of reduced physical activity may have deleterious effects on many organs and systems, and these effects might be more pronounced and more challenging to reverse in certain more vulnerable populations, such as the chronically ill and the elderly.

## Acute and sustained effects of physical activity

Regular moderate physical activity has wide reaching health benefits for people of all ages, sexes, races, health conditions and shapes, as shown in reduced morbidity and mortality rates, increased quality of life and independence in old age [6]. Physical fitness can also help reduce the risk of acute life threatening events [14, 15]. To maintain these effects sustained, optimally lifelong physical activity is required, since acute benefits are transient and dissipate over time, unless physical activity stimulus is repeated. However, knowledge about acute impact of single exercise bouts might facilitate the motivation and communication with patients to take up more activity now. The current crisis could potentially be a window of opportunity, a learning moment to initiate long-term activity.

## Acute metabolic and vascular effects of physical activity

Physical activity exerts a major influence on human metabolism. Physical activity acutely increases glucose uptake, thus lowering circulating blood glucose level. This uptake by contracting skeletal muscles takes place through insulin independent mechanisms [16]. A single exercise bout also induces beneficial metabolic effects after exercise. Muscle insulin sensitivity is increased for up to 48 h after exercise in healthy individuals [16].

Even relatively low volume of simple physical activity, such as walking or cycling has been shown to induce favorable effects on various metabolic markers in healthy and diseased population. As little as 15 min of post-meal walking may blunt the glycemic response in healthy women [17] and in women at risk of diabetes [18]. Takaishi et al. found that 6 min of another easily available physical activity, stair climbing and descending sufficed to reduce post-prandial glucose levels in inactive middle aged men with impaired glucose tolerance [19].

The potential of both aerobic and muscle strengthening physical activity to lower postprandial lipidemia is well established [20]. For example, in young healthy participants as little as 3 times 10 min of brisk walking a day reduced triacylglycerol concentration (area under the curve for plasma concentrations) by 16% [21].

A single bout of physical activity is followed by an acute decrease in blood pressure, known as post-exercise hypotension. A current systematic review based on 65 individual studies found that physical activity, regardless of participant and exercise characteristics, leads to a clinically relevant reduction in blood pressure [22].

## Acute effects on the immune system

Inflammatory processes have been linked to chronic diseases with the largest disease burden, such as cardiovascular diseases, type 2 diabetes, cancer, dementia, osteoporosis via irreversible organ damage and dysfunction [23]. A systematic review concludes that a single bout (30–60 min) of moderate to high intensity physical activity can increase the activity of both circulating IL-6 and neutrophil counts in untrained adults [24].

## Acute effects on mental health, wellbeing, mood, sleep and cognition

As stated above, it is reasonable to expect that the lockdown exerts unfavorable psychological effects on the short and possibly longer term. Because of the well documented acute impact of physical activity on symptoms of psychological distress [25] and potential to increase positive activated affect [26] physical activity should be recommended as a nonpharmacological countermeasure. Beyond improved positive affective physical activity can also promote feelings of vitality [27]. Importantly, even low to moderate volume and low to moderate intensity exercise seems to be beneficial [26]. Indeed as little as 10 min moderate intensity walking can improve mood [28].

Also strong meta-analytic evidence demonstrates the anxiolytic effect of acute relatively short (20 to 30 min) activity bouts in adults and older adults [29]. Physical activity, irrespective of intensity and time of day at which activity is performed, can acutely improve various sleep outcomes [29]. Single short (10–20 min) physical activity bouts lead to improved cognition, with most consistent results for domains of executive function [29].

## What should we recommend?

We have shown that on the one hand even short term reduced physical activity may exert harmful effects on physical and mental health and on the other physical activity can acutely counteract exactly these effects. In the following we provide guidance on pragmatic, evidence based physical activity programs that are feasible also under current situations including lockdown.

Whenever and wherever permitted and possible, while complying with local and national regulations, people should be encouraged to be active outdoors, preferably in green areas. As in all other situations, rules of social distancing (at least 1,5–2 m or six feet) are essential also outdoors. The use of surgical or homemade masks in the community setting has been discussed in a controversial manner. Whereas the Center of Disease Control recommends them [30], the World Health Organization maintains that “the wide use of masks by healthy people in the community setting is not supported by current evidence and carries uncertainties and critical risks” [31]. It has to be considered that masks might impede airflow and therefore provoke discomfort especially with more strenuous exercise. Limited air exchange around the face can lead to a warm, humid microclimate. Mask use and disposal make certain safety measures necessary [31].

Possible outdoor activities include (brisk) walking, jogging and cycling. Green exercise can improve wellbeing [32], and sensible sun exposure might boost the immune system [33]. Novice exercisers should start with low volumes (5–10 min 2–3 times and week) and low intensity (regular speed) and increase both volume and intensity with time. Alternating short periods (e.g. 3 min) with lower and higher speeds can enhance health effects and make the workout more enjoyable [7].

If leaving one’s house is not permitted, staircases can be used for exercising the lower extremities. Depending on the speed, staircase walking can also function as a cardiovascular exercise. People with compromised balance should hold on to the rails, in this case use of gloves is recommended and washing hands obligatory.

A large number of exercises for improving cardiovascular and muscular fitness, as well as balance can be performed even in very small spaces without devices. Walking in place or around the apartment, jumping and hopping are such options. Similarly to walking outside, volume and intensity should be increased only gradually.

Muscle strengthening exercises can be carried out using elastic bands, if available [34]. If not, exercises, such as sit up, push up, squats etc., all using nothing but one’s own body weight, are a perfect option. All these exercises can be modified to accommodate for less well trained or elderly people [35]. Various national and international health and sports agencies provide further ideas and examples of home-based exercises [36].

New evidence suggests that physical activity bouts can be short (also less than 10 min) and time spent in activity can be accumulated throughout the week [6]. Starting with shorter bouts and building up activity time gradually might be more accessible for currently inactive people.

To limit adverse events, no physical activity should be performed during acute infection (also other than COVID-19) and in case of absolute contraindications [37]. We do not recommend excessive exercise (high volume high intensity training), since it can lead to transient states of immunodepression and increase of susceptibility to infection [38].

## Conclusion

Lockdown implemented in an attempt to contain the SARS-CoV-2 virus is unprecedented for most Western countries, and represents a major societal challenge with conceivable repercussions for people’s mental and physical health. Physical activity has the potential to ward off detrimental cardiometabolic effects of inactivity and to strengthen psychological resources and coping skills. Physicians and other health care professionals should use this time as a window of opportunity to provide physical activity counseling to their patients.

## Data Availability

All data generated or analyzed during this study are included in this published article.
